# Improving conservation strategies of raptors through landscape ecology analysis: The case of the endemic Cuban Black Hawk

**DOI:** 10.1002/ece3.5815

**Published:** 2019-11-27

**Authors:** Yarelys Ferrer‐Sánchez, Ricardo Rodríguez‐Estrella, Miguel Ángel Martínez‐Morales

**Affiliations:** ^1^ Universidad Técnica Estatal de Quevedo Quevedo Los Ríos Ecuador; ^2^ Centro de Investigaciones Biológicas del Noroeste La Paz Baja California Sur México; ^3^ School of Natural Resources and the Environment University of Arizona Tucson AZ USA; ^4^ Departamento de Conservación de la Biodiversidad Unidad San Cristóbal El Colegio de la Frontera Sur San Cristóbal de las Casas Chiapas México

**Keywords:** *Buteogallus gundlachii*, Cuban Black Hawk, ecological niche modeling, human activity, landscape analysis, nest‐site selection, protected areas design

## Abstract

1. Raptor species conservation should consider a landscape perspective in order to include habitat requirements associated to large home ranges around nesting sites. Landscape analysis can help to better understand raptor habitat requirements and the degree of tolerance to habitat changes at different scales.
2. We used a landscape ecology perspective to determine the nesting habitat selection of endemic and endangered Cuban Black Hawk, and using ecological niche modeling, we obtained the potential distribution of nests to evaluate the effectiveness of protected areas (PAs) for raptor conservation.
3. Nesting habitat selection was related to breeding success at a landscape scale using data from 27 different nesting sites during 2012–2013 breeding seasons. The potential nesting areas distribution was compared with current officially PAs design in the central region of Cuba.
4. All nests were located in mangrove swamp. Pairs chose nesting sites with low soil–vegetation moisture and low soil reflectance. At the landscape level, they selected low shape complexity of patches and few patches of coastal vegetation around nesting sites which contained similar mangrove patch size and shape. The potential distribution of nests increased close to the coastline. The model predicted a suitable narrow area of 556 km^2^, and the most favorable nesting area represented 2% of this total. 33% of nests were located within officially natural protected areas while 27% were close to or inside highly threatened areas. A 16% of high to medium suitable nesting habitat overlaps with urban areas. Currently, PAs contain 23% of the nesting area distribution.
5. Our study shows landscape ecology and nest‐site selection approach is crucial to evaluate the persistence of Cuban Black Hawk, as environmental variables and human activity can be related to its productivity. This approach can be applied in conservation strategies of island raptors.

## Graphical abstract

A landscape ecology perspective was used to determine the nesting habitat selection and breeding success at different scales of endemic and endangered Cuban Black Hawk. All nests were located in mangrove swamp in sites with low soil–vegetation moisture and low soil reflectance, and at the landscape level they selected low shape complexity of patches and few patches of coastal vegetation. The model predicted that the most favorable nesting area represented 2% of total suitable habitat, and that 33% of nests were located within officially natural protected areas, thus these areas do not efficiently protect Cuban Black Hawks.

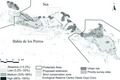

## 1 INTRODUCTION

Habitat selection defines the distributional patterns of birds (Cody, 1985; Janes, 1985), as well as their biogeographical patterns (Rangel, Diniz‐Filho, & Bini, 2006), and refers to a hierarchical process of responses that may result in a disproportionate use of particular habitats to enhance survival and fitness of individuals (Hutto, 1985). Nesting site selection refers to habitat selection for breeding (Jones, 2001) and is critical for birds as selected sites likely influence their reproductive success. This process may be similar throughout the species distributional range, or it may vary depending on optimal–suboptimal habitat availability and constraints on nesting success among regions (Gjerdrum, Elphick, & Rubega, 2005). Understanding the determinants of nesting site selection and their consequences for nesting success is a complex issue, but still urgently needed to understand the evolutionary responses of species. Generalists species seem to be more tolerant to habitat gradients and changes than specialists that tolerate less habitat variation (Devictor, Julliard, & Jiguet, 2008; Ferrer‐Sánchez & Rodríguez‐Estrella, 2015). At present, when habitat loss is one of the main causes of species extinction and of the dramatic loss of biodiversity in all ecosystems throughout the world (Ceballos et al., 2015), understanding the process of habitat selection is critically important for species conservation. It is still more relevant for vulnerable populations and for endemic and rare species.

Interpretations of habitat selection should consider temporal and spatial scales on which they are investigated (Wiens, 1986). Traditionally, studies of habitat selection have focused on a fine scale (e.g., microhabitat) whereas the processes underlying observed patterns (e.g., nest‐site selection, foraging and mating behaviors) may actually take place on a much broader scale (Wiens, 1995), usually drawing incorrect conclusions regarding habitat requirements. For instance, landscape variables may be able to explain effects that in the past have been attributed to patch level features (Lee, Fahrig, Freemark, & Currie, 2002). In addition, elements of landscape heterogeneity can influence a variety of ecological responses, including animal movement (e.g., Fahrig, 2007), population persistence (Fraterrigo, Pearson, & Turner, 2009), and species interactions (Polis, Power, & Huxel, 2004). The spatial configuration of the habitat and habitat quality affects breeding success, thus this is an issue increasingly associated with landscape ecology (Flather & Sauer, 1996; Suárez, Balbontin, & Ferrer, 2000).

In general, raptors‐habitat associations seem to respond to the landscape at multiple scales (e.g., Šálek et al., 2016). However, most studies concerning habitat selection by raptors focus on microhabitat variables such as tree characteristics, ground cover or perches, often measured at small‐detailed scales (Sánchez‐Zapata & Calvo, 1999). The incorporation of habitat data at the landscape level has resulted in better approaches even using a metapopulation perspective (Sánchez‐Zapata & Calvo, 1999), and it is crucial in studies for conservation of rare and endangered species (e.g., Gastón et al., 2017; Mateo‐Sánchez et al., 2016). Few studies have evaluated the landscape level at more than one scale for species with large home ranges and for top‐order predators (Wallace, Kennedy, Squires, Olson, & Oakleaf, 2016); predators select habitat features at multiple spatial scales (Sergio, Pedrini, & Marchesi, 2003). Thus, conservation of viable populations of predators requires integrated researches, planning, and management at the landscape level (Pedrini & Sergio, 2002) and should consider multiple spatial scales.

Many Neotropical raptors are considered uncommon or rare species, and the current status of a great percentage of them is of special concern because many will become rarer in the medium term (Ferrer‐Sánchez & Rodríguez‐Estrella, 2016), it has been estimated 46% are threatened by human transformation of native ecosystems (Sarasola, Grande, & Bechard, 2018). Furthermore, there is particular concern on the conservation of rare endemic island raptors because they are highly prompt to extinction, mainly those with a narrow distribution, narrow habitat tolerance, and small population size (Gaston & Fuller, 2009). In studies of Neotropical raptors, and particularly of rare endemic island species, landscape ecology approaches are crucial to evaluate the effects of environmental variables and human activity on nesting site selection and productivity of vulnerable populations.

**Figure 1:**
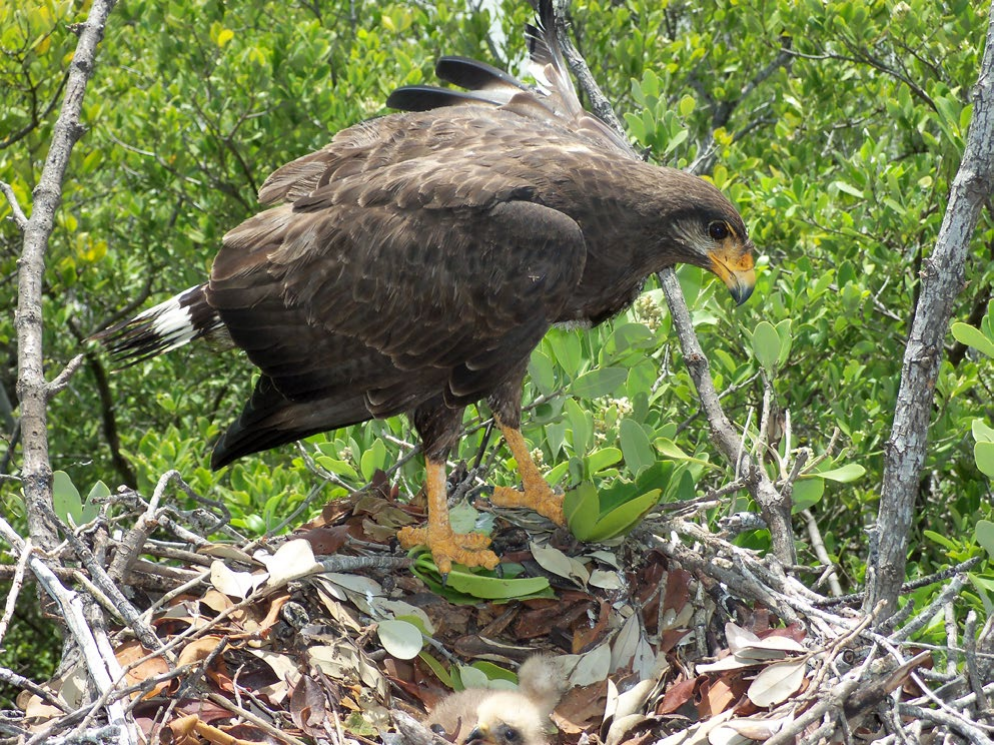
The Cuban Black Hawk is an endemic diurnal raptor in Cuba, inhabiting the edge of coastal wetlands and marshes, salt marshes, beaches, mangroves, and mesophilic semideciduous forests surrounding coastal areas. Its habitat has been highly fragmented, and its population size is unknown, it is listed as endangered in Cuba due to tourism development

The Cuban Black Hawk (*Buteogallus gundlachii*) is one of the three endemic diurnal raptors in Cuba (Figure 1). It inhabits the edge of coastal wetlands and marshes, salt marshes, beaches, mangroves, and mesophilic semideciduous forests surrounding coastal areas (Rodríguez‐Santana & Viña, 2012). The Cuban Black Hawk has specialized habits and a restricted distribution (Ferrer‐Sánchez & Rodríguez‐Estrella, 2016), feeding mainly on crabs (Wiley & Garrido, 2005). About 50% of its habitat has been fragmented in the last 100 years, and its population size is unknown (Rodríguez‐Santana & Viña, 2012). At present, little is known about its natural history and reproductive biology, having poor descriptions of nests and the breeding season (e.g., Valdés Miró, 1984). In general, detailed quantitative information on the population status, distribution, feeding, and reproduction of this raptor is lacking, preventing to propose and promote better conservation actions (Ferrer‐Sánchez & Rodríguez‐Estrella, 2016).

In general, all endemic raptors in Cuba are endangered due to habitat loss and fragmentation, deforestation, hunting, agriculture, ranching, and urbanization (Rodríguez‐Santana & Viña, 2012). Specifically, the Cuban Black Hawk faces a strong threat by tourism development because hotel infrastructure is covering almost all the distributional range of the species along the Cuban coastline and the larger archipelagos, destroying and modifying its main habitat. The Cuban Black Hawk is considered as a Near Threatened species by the 2017 IUCN red‐list. Nevertheless, a potential distribution model for this species predicted a very narrow geographic distribution, mainly in the mangrove swamp. Forty‐five percent of the Cuban Black Hawk geographic distribution occupies 45% of the mangrove area (Ferrer‐Sánchez & Rodríguez‐Estrella, 2016). The current conservation status of the Cuban Black Hawk, the small population size, and its particular ecological requirements make the species highly vulnerable, and thus, this is a priority species for conservation.

The Sabana‐Camagüey archipelago covers most of the Cuban Black Hawk population and is the second most important Cuban touristic spot. Certainly, the limited ecological and biological information for this raptor precludes any proper conservation and management plan for the species. For instance, studies of reproductive rates, nesting success, and productivity can be valuable in assessing its population status and to understand the limiting environmental factors for the species. Data on reproduction can help predicting the effects of land use changes on the Cuban Black Hawk nesting grounds and on the population dynamics. Studies on nesting site selection may help managers to identify habitat characteristics and important areas for the Cuban Black Hawk at a landscape level. A landscape approach will help to have better and more efficient conservation strategies for this endemic and endangered species in Cuba.

The main objectives of this study were as follows: (a) to characterize for the first time the nesting site features, nesting success, and productivity of the Cuban Black Hawk; (b) to compare the characteristics of the nesting sites with the features of the available habitat to determine habitat selection through a landscape ecology perspective; (c) to model the ecological niche in order to get the potential distribution of nests; and (d) to evaluate the effectiveness of protected areas (PAs) in terms of raptor conservation in order to better inform decision‐making for PAs. Our prediction is that the network of current protected areas is not covering the total nesting area of the endemic Cuban Black Hawk. Most of the methodological and conservation problems that rare, endemic raptors face on the island of Cuba are common to all 97 Neotropical raptor species. Thus, our approach to assess habitat selection and productivity at the landscape level for the Cuban Black Hawk to improve decision‐making and conservation will also be useful for other raptor species, particularly those that are rare and endangered on islands.

## 2 METHODS

**Figure 2:**
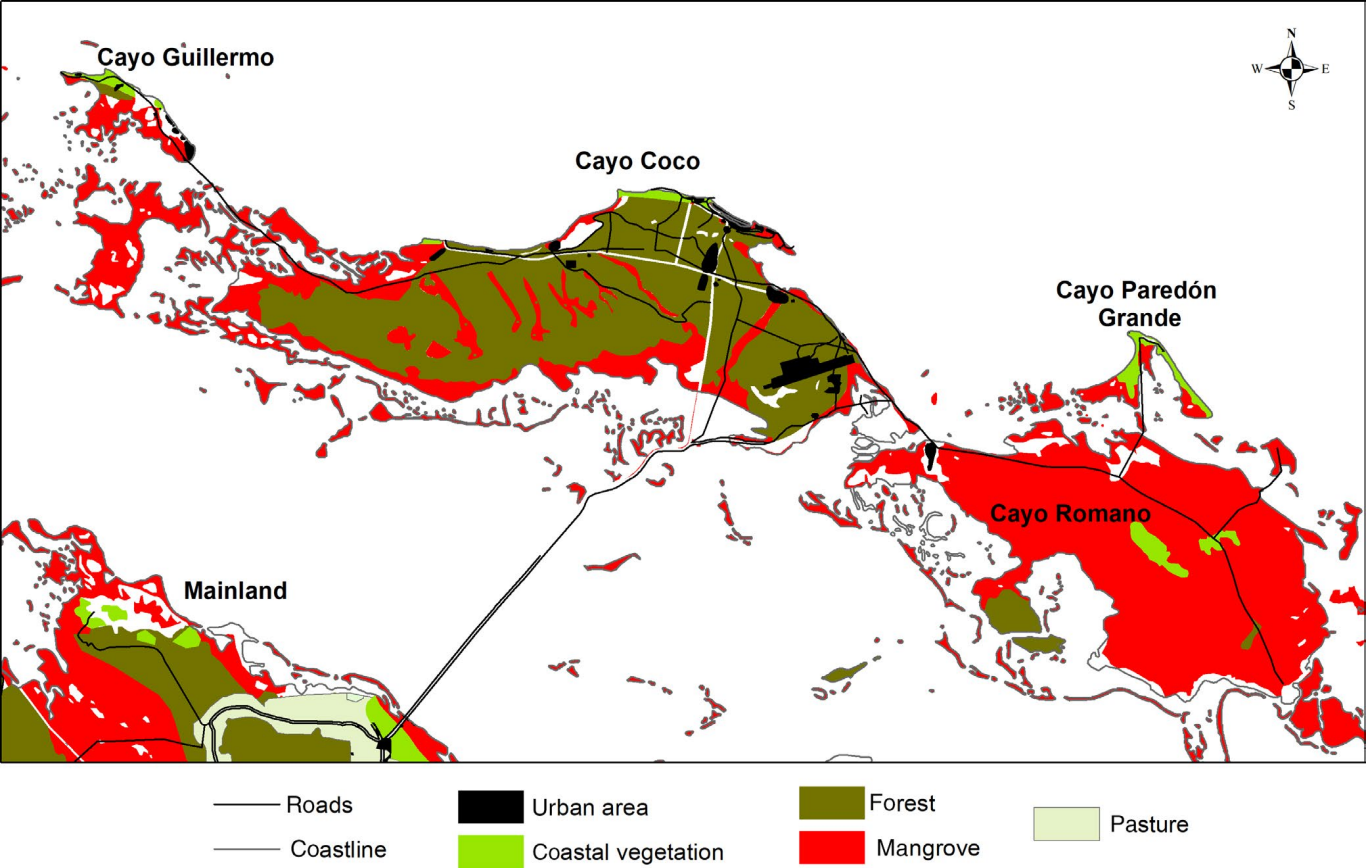
Land use and vegetation types in the study region, Ciego de Ávila, Cuba, during 2012–2013 Cuban Black Hawk nesting seasons

Fieldwork was carried out in the Sabana‐Camagüey archipelago in the central region of Cuba (863.3 km^2^; 22.514844°, −78.431758°), at the Gran Humedal del Norte de Ciego de Ávila Ramsar site (Figure 2). Sampling sites included the cays Guillermo, Coco, Paredón Grande, and a part of Romano. Cays along the coastline of Cuba have a diversity of vegetation types such as mangroves, xeromorphic coastal shrubs, semideciduous forests, microphyllous evergreen forests, halophytic vegetation, and plant communities of rocky and sandy beaches. This vegetation is similar in all the distributional range of the Cuban Black Hawk; therefore, our results have application along the country. A more detailed description of the area can be found elsewhere (Ferrer‐Sánchez & Rodríguez‐Estrella, 2015).

Encroaching tourism development has reduced the distribution of mangroves. Since 1988, a roadway constructed between the cays and main island has destroyed large areas of mangroves. This construction did not take into account the ocean current circulation and the high biodiversity zones. Some of the coastal areas have had strong impacts because of their use as a quarry for gravel mining and the construction of roads and facilities for tourism. The tourism development has partially reduced by 70% the extent of native vegetation (Ferrer‐Sánchez & Rodríguez‐Estrella, 2015). Most of the land in the cays is used for tourism, with PAs at the western portion. This region is one of just a few wide cays in Cuba where the Cuban Black Hawk breeds in high densities.

### 2.1 Nest survey and reproductive parameters

To locate nests, we surveyed all accessible habitats on foot and from vehicle during the 2012 and 2013 breeding seasons (February–June). We determined nest locations by following adults flying to nests and by visual inspection of potential nesting sites. Locations of nests were registered with a GPS.

The evidence that a nesting pair occupied a territory was based on observations of two birds that appeared to be paired, or one or more adults engaged in territorial defense, courtship, and presence of eggs or young. In addition, we recorded the presence of a nest whenever it had been recently built, repaired, or decorated as an evidence for territorial occupancy. A nesting pair was defined as a pair occupying a nest, and a laying pair was defined as a pair that laid eggs (Steenhof & Newton, 2007). If the same nest site was used in consecutive years, we only included the data of the first year in the analyses of nesting sites. To minimize the risk of nest abandonment, we visited each nest only 2–3 times during the breeding period. The number of fledglings was determined for accessible nests. A nest was considered successful if nestlings were recorded 1 week before the expected fledging date or when fledglings were detected in the area surrounding the nest (Sadoti, 2008). We estimated the proportion of laying pairs/nesting pairs, clutch size per nest, hatching success (number of nestlings/number of eggs laid), fledging success (number of fledglings/number of nestlings), productivity per active nesting pair (number of fledglings/active nesting pair), and productivity per successful pair (number of fledglings/successful nest).

We measured nest‐site characteristics only after the fledglings had abandoned the nest or when the nest failed. We recorded the tree species and measured the height of trees and nests, if dead or alive and the proportion of canopy coverage. We estimated land cover of the area surrounding each nest within a radius of 2 km using a GIS. The radius was based on the overall linear distance traveled by *Buteogallus anthracinus* during the incubation period and after fledging (Schnell, 1994), because there is no information on its habitat use, distance traveled away from nest and home range. Finally, we estimated nest density. To estimate the nest density, we considered only nests in suitable habitat (e.g., mangrove) for nesting. Considering an estimation that included nonsuitable habitat (e.g., forest or pasture) would produce a density even smaller and would be erroneous from an ecological perspective.

### 2.2 Landscape variables

Nests were marked on maps (1:50,000 land use and vegetation map). We selected the landscape variables based on their potential influence on the nesting preferences of the species. We then estimated their availability using digital cartography and the representativeness of these habitats in the study area. We generated variables to describe landscape heterogeneity and patchiness using a vegetation and land use map created by us. We developed a vegetation and land use map to identify human‐modified areas and several classes of natural vegetation. A supervised classification of two multispectral Landsat ETM + images of the study area (date: April 12, 2012; Projection UTM–Datum WGS84, 30 m spatial resolution) was used. Both images were overlapped with a mosaicking algorithm. Thirty‐eight training polygons were digitized for each of the eight coverage classes (i.e., mangrove, forest, coastal vegetation, lagoon, swamp marsh, cattle pastures, agriculture, and urban areas) to develop spectral signatures to guide the classification. We then classified the entire image using a maximum likelihood algorithm (Richards, 1986). As a basis for supervised classification, greenness, brightness, and wetness bands obtained by the Tasseled Cap method were also used. Image processing and analyses were made in ENVI 5.0 (Exelis VIS) (Figure 2). Metrics describing landscape spatial structure and heterogeneity were derived from a grid patch analysis at class and landscape level using PatchGrid extension in ArcView 3.2 (ESRI Inc.). A pixel size of 100 m was defined considering the average size of the smaller vegetation patches in which the Cuban Black Hawk was sighted.

Metrics that can influence the nesting preferences of a species were selected (Oja, Alamets, & Pärnamets, 2005; Reiley & Benson, 2019). Metrics included patch density and size, shape, diversity, interspersion, and landscape metrics as a measure of habitat heterogeneity and complexity (McGarigal & Marks, 1995). Mean shape index measures the average patch shape (average perimeter‐area ratio), for a particular patch type (more geometrically complex with more habitat edge; McGarigal & Marks, 1995). We measured the distance from nests to edge of patches and to the coastline. To measure the distance to the water of both nests and points without nests, the vector map of the coastline of Cuba was used. This spatial analysis was carried out through maps in a GIS, not in situ, in such a way that the errors associated with the mapping affect both the measurements from the nests and from the points without nests. In addition, we believe that the estimated distance we used has a good approximation to the real one, especially knowing that up tides and low tides have little variation in Cuba, between 0.27–0.39 m and 0.09–0.12 m, respectively. In order to assess human disturbance, we measured the proximity of paved roads and human activity infrastructure to each nest and calculated paved road density using *Nearest features* and *Drainage*/*Lineament*/*Road*/*Density* extensions.

We considered a disturbance index as the urban area contained in the radius of 2 km from the nest. Furthermore, we developed a qualitative scale to associate nests with habitat human disturbance: (a) nests surrounded by undisturbed natural vegetation; (b) natural vegetation is well preserved, but there are evident habitat alterations (3%); (c) around 5%–10% of habitat alteration; (d) >10% of habitat alteration. This qualitative scale was used to obtain easy presentation results and easy understanding in order to propose better nesting habitat management actions.

### 2.3 Nesting site selection

Habitat selection analyses require a comparison of selected sites (presences) with a randomly chosen control set of nonused sites (pseudo‐absences) (Manly, McDonald, & Thomas, 1993). In order to compare the features surrounding nests against the available environment with potential features to nest, we generated 108 random plots (four plots per each nesting site). Plots located within 100 m from other random plots or nest were excluded from the analysis. This was the minimum within‐year interpair nest distance observed in this study. Also, we excluded those plots located in urban areas or forest (beyond 100 m from the coastline) because these sites are not used as nesting sites by the raptor. For each random plot, we measured the same variables as for the nesting sites (see Section 2.2).

### 2.4 Statistical analyses

We examined pairwise Spearman correlation coefficients among 58 variables (Appendix S1). For correlations |*r*| > .7, only the most ecologically relevant variable was retained for further analyses (Dormann et al., 2013). Through this procedure, the original set of variables was reduced to 32. We used the nonparametric Mann–Whitney *U* test to compare the features of nests and random plots (landscapes).

Principal component analysis (PCA) is often used to reduce data and stabilize subsequent statistical analyses (Vaughan & Ormerod, 2005) and also because the PCA reduces collinearity and yield better ecological models (Dormann et al., 2013). A PCA was applied including the 32 variables to assess the statistical dimension of the structure of landscapes and to define a set of core variables. By using orthogonal varimax rotations of the axes, we identify clusters of collinear variables (i.e., groups of variables with high loadings on the same principal component and extracted from each cluster the single variables with highest loadings (Dormann et al., 2013). We retained components by using the criteria of cumulative variance (up to 50%) and the eigenvalues that should be >1.0. For each retained component, the variables with absolute loading >0.7 were considered associated with the component. Posteriorly, we analyzed the relationships between the presence of nests and the set of components of the PCA by means of a logistic regression, through a generalized linear model (GLM) procedure using a logistic link function and a binomial error, to identify the set of variables that best separated nest sites from random sites (Jongman, ter Braak, & Van Tongeren, 1995). In this case, we used components as variables in the logistic regression.

Given the data and a set of candidate models for the underlying process (e.g., nesting habitat selection), that combine variables and its interactions, we wanted to determine the model that better approximates the likely “true” process. Interactions between variables were included to identify if some trend among data existed and then finding a plausible ecological explanation. For example, we related the wetness and soil brightness with the landscape shape and the number of coastal vegetation patches since we hypothesized that irregular‐shaped landscapes might have more bare ground and more variability in wetness and brightness with respect to landscapes with a more regular shape. Furthermore, a greater number of habitat patches (coastal vegetation) could have a greater variability in wetness and soil brightness. Thus, this greater variability in wetness and brightness could be related to a better access and availability to water and preys.

Using a forward stepwise procedure, each component (variable) was tested for significance in turn, and the variable contributing to the largest significant change in deviance from the null model was then selected and fitted. At each step, the significance of the variables included in the model was tested and any falling below the criterion level of *p* = .05 was excluded. The final model was considered to have been identified when all the variables had a significant effect at *p* < .05. We ranked models using the Akaike information criteria (AIC), and the Bayesian information criterion (BIC)/Schwarz Criterion value (Burnham & Anderson, 2002; Fabozzi, Focardi, Rachev, & Arshanapalli, 2014); the best model had the least weight for both measures (Wagenmakers & Farrell, 2004). Data were analyzed using the software R Core Team (2015).

### 2.5 Ecological niche model

#### 2.5.1 Data treatment

Having obtained the environmental variables associated with the first five components of PCA (those with the highest weight ≥0.8 per component), we generated data layers (100 m pixel resolution which we considered adequate for this raptor) of the variables using the modules PATTERN, PATCH AREA, AREA and CRATIO in Idrisi Selva (Clarks Lab). For example, the PATTERN module computes various numerical pattern indexes (relative richness, diversity, domain, frequency, fragmentation, and others) using a 3 × 3, 5 × 5, or 7 × 7 window. The PATCH AREA module groups the adjacent pixels of similar land surface category into patches, calculates their areas, and produces an image where each pixel corresponds to the area of the patch to which it belongs. We masked these layers to the extent of a polygon of the cays that represents a hypothetical area of historical accessibility of the species in the region (M area; Soberón & Peterson, 2005). We used a vectorial map of land use and vegetation generated previously (Ferrer‐Sánchez & Rodríguez‐Estrella, 2015) to determine the M area. Moreover, we reduced collinearity among environmental layers analyzing pairwise Spearman correlation coefficients for each of the variables within the M area. For those variables that showed a correlation above .8, we removed all except the variable considered the driver of the most variation in the predictor variable set as determined by the former PCA. Removing highly correlated predictor variables from Ecological Niche Models can increase model performance (Cooper et al., 2016).

On the other hand, occurrence records are commonly grouped in areas with greater sampling effort, which may result in spatial autocorrelation. Numerous studies have used spatial filtering to avoid spatial autocorrelation by excluding records very close to each other (Boria, Olson, Goodman, & Anderson, 2014; Machado et al., 2019). Spatial filtering reduces overfitting effects, which occurs when the model is too tightly adjusted to calibration data and cannot predict data accuracy independently (Radosavljevic & Anderson, 2014). All nest locations were then carefully checked, filtering their presence in a 100 m pixel size as previously defined.

#### 2.5.2 Model construction

Using the environmental layers and the occurrence data of each nest, we built an ecological niche model for nesting sites by means of the maximum entropy algorithm (Maxent 3.4.1) (Phillips, Dudík, & Schapire, 2018), estimating environmental suitability and the potential distribution of nests. Among presence‐only data algorithms, Maxent has one of the better predictive abilities (Elith et al., 2011) and has strong robustness to small sample sizes (Wisz et al., 2008). We used the default parameter for beta multiplier values and feature classes. Calibration data were used across the accessible area (M) with the Maxent bootstrapping/replicated settings and a “cloglog” output. To reduce uncertainty due to sampling, and to explore effects of specific calibration data sets on model outputs, we ran 100 replicates of models with a random seed partition and a bootstrap replicate type. Of the 100 replicates, those that had a calibration AUC >0.9 were chosen (16 models). Of these 16 selected models, the most important variables were chosen (>50% added or individually), and among these, three variables were chosen with little correlation (*r* < .7) and related to the species ecology. In this way, the humidity, relative richness and variability of mangrove patch sizes were chosen as the predictive variables in the final model, for which 100 replicates were also run. Among the 100 replicates, the median model was selected avoiding the effects of outliers.

Finally, Maxent output was converted into binary map using the minimum training presence threshold value (Liu, Berry, Dawson, & Pearson, 2005). All pixels with a value under this threshold were assigned a value of zero (0), which would represent absence of nests. We know that often threshold predictions reflect the assumptions of researcher about appropriate threshold values and not the attributes of the species distribution (Merow, Smith, & Silander, 2013). Nevertheless, we considered this threshold better than others for nests distribution because the Cuban Black Hawk is a habitat specialist and has a restricted distribution. Modeling the distribution of rare and endangered species is challenging. Reducing omission errors is the most important determinant of threshold selection method, and this was achieved with the minimum training presence threshold.

The difference between the cut threshold value (minimum training presence: 0.032) and the maximum probability value was divided by three, yielding a new cut value that was used to classify the map into three new categories. These categories include the distribution of pixels above the threshold value (0.032): low (3.2%–33%), medium (33%–66%), and high probability (66%–100%) of favorable conditions for nesting.

#### 2.5.3 Model evaluation

We evaluated the predictive performance of models calculating the AUC_ratio_ in partial ROC (receiver operating characteristic) analyses using the NicheToolBox R package (Osorio‐Olvera, 2016), with 0.05 as the proportion of omission, 50% of random points, and 10,000 iterations for the bootstrap. This technique is based on the traditional ROC (Fielding & Bell, 1997), but considering the coverage area of the commission error axis by model predictions, and giving preference to omission over commission error in the evaluation of model strength (Peterson, Papeş, & Soberón, 2008). AUC ratio values above 1 indicate that models outperform the null expectation (Peterson et al., 2008).

### 2.6 Anthropogenic risk assessment

In order to assess the anthropogenic risk on the nesting sites, the urban and road layers were overlaid with the potential distribution of nesting sites. Buffers of 500 m around the urban areas and 100 m around the paved roads were generated. The suitable habitats (according to the MaxEnt predictions) located inside the buffers and outside PAs were classified as at high anthropogenic risk because of a lack of any protection. We selected a distance of more than 500 m apart from the human access points (roads or hotel infrastructure) as a conservative distance threshold. We selected more than 500 m as a distance apart from the human access points as a conservative distance threshold because it can be taken as a proxy of human access to the countryside in these cays. In this area, there are no strong human activity outside hotels, roads, or other infrastructures. Also, coastal vegetation, forests, lagoons, and mangroves are around the hotels and infrastructures, and they are very dense, hard for humans to access these habitats beyond 400–500 m; therefore, anything beyond this distance was considered at low risk. We calculated percentages of suitable habitat at high risk and identified vulnerable nesting sites.

### 2.7 Priority areas for conservation

We assessed the extension and proportion of the potential distribution of the nesting sites within the official protected areas network. We evaluated the feasibility to increase the boundaries of the current PAs or to re‐evaluate its conservation zones according to the favorable conditions probabilities for the nesting sites that were outside of official protection. Having this information, we proposed a set of priority survey areas. We restricted our consideration of PAs to those with established management plans.

## 3 RESULTS

### 3.1 Nests

We located 18 nest territories in 2012, four in cayo Guillermo, nine in cayo Coco, two in cayo Romano, and three in cayo Paredón Grande. In 2013, we found nine new territories and three territories that were reoccupied. All nests were located in mangrove swamp except one nest located in semideciduous forest. Our landscape analyses considered 27 different territories.

Most nests were built below the canopy in trees of *Avicennia germinans* (48.5%) and *Rizophora mangle* (39.4%). All trees containing nests (*n* = 27) were alive and mature. The nests mean height was 5.2 ± 2.0 m (range: 3–10 m), whereas trees had a mean height of 7.8 ± 2.2 m (range: 5–12 m, *n* = 27). Trees had a mean DBH = 0.6 ± 0.5 m (range: 0.3–2.9 m). The canopy coverage was 51.1 ± 29.1% (10%–100%), and the nests mean distance from water surface was 54.6 ± 85.9 (0–200 m).

The mean internest distance for pairs breeding in 2012 was 24.0 ± 18.6 km (0.09–55.4 km, *n* = 18), and the average distance to the nearest nest was 1.6 ± 2.1 km (0.09–8.8 km, *n* = 18). In 2013, the mean internest distance was 20.9 ± 14.6 km (0.36–50.7 km, *N* = 12) and the nearest nest was 1.8 ± 2.6 km (0.36–10.5 km). No between‐years differences were detected (*p* = .1). Nest density including both years was 0.035 nest/km^2^ (or 3.5 nests/100 km^2^).

**Figure 3:**
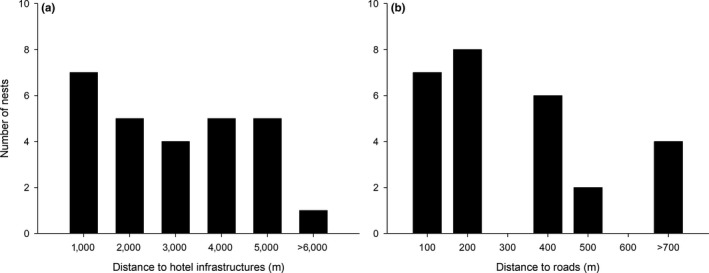
Number of Cuban Black Hawk nests in relation to distance to hotel infrastructures (a) and roads (b) in Ciego de Ávila, Cuba, during 2012–2013 nesting seasons

In total, 63% of nests (*n* = 17) were located in areas with undisturbed native vegetation and at least 1 km away from tourist buildings (Figure 3a). None of the nests were located in areas with more than 10% of habitat alteration, 15% of nests were in well preserved native vegetation with 3% of habitat alteration, and 23% were located in native vegetation with around 5% of habitat alteration. More than 50% of nests were close to paved roads (Figure 3b).

### 3.2 Reproductive parameters

The proportion of laying pairs as a function of nesting pairs was 0.8. Eggs were laid from March 19 to June 18. A great proportion of pairs (39%) laid eggs on May (2–10). Forty‐one eggs were laid with an average clutch size of 1.6 ± 0.5 eggs (range: 1–2 eggs; nests = 27). We did not record eggs in 21% of the occupied nests, but we could not know whether these nests had failed prematurely; that is, before egg laying, or whether eggs had been laid and subsequently lost. The hatching success was 60% with 1.3 ± 0.5 nestlings/nest. The fledging success was 80%. The productivity per total active nesting pairs was 0.7 ± 0.3, and 1.2 ± 0.1 fledglings/successful nest.

### 3.3 Nest‐site selection

A high number of combinations of environmental variables (161) were significantly correlated (Spearman rank correlation, *p* < .0001). Nine out of 32 variables differed between nesting sites and random plots (*U* test, *p* < .05; Table 1). Nesting sites exhibited shorter distances to paved roads, urban areas and coastline, greater distances from patch edge, a higher density of roads, smaller forest patches, larger total forest and coastal vegetation areas, and more lagoons than random plots (Table 1).

**Table 1:** Characteristics of the Cuban Black Hawk nesting sites and random plots in the Gran Humedal del Norte de Ciego de Ávila, Cuba, during 2012–2013 nesting seasons

Variable	Nesting site (*n* = 27) Mean (*SD*)	Random plot (*n* = 99) Mean (*SD*)	*U* test	
			*U*	*p* Value
Distance to road (km)	0.32 (0.34)	2.90 (2.70)	243	*<.0001*
Distance to human activity (km)	2.52 (1.84)	6.27 (5.33)	702	*<.0001*
Distance to coast (km)	0.24 (0.20)	2.0 (2.70)	599	*<.0001*
Distance to patch edge (km)	0.53 (0.41)	0.16 (0.36)	2,118	*<.0001*
Density of roads	0.1 (0.0)	0.04 (0.04)	2,377	*<.0001*
Optimized soil‐adjusted vegetation index (OSAVI)	−0.2 (0.2)	−0.2 (0.3)	1,292	.79
OSAVI *SD*	0.4 (0.1)	0.4 (0.1)	1,499	.33
Wetness (interrelationship of soil and canopy moisture)	−35.5 (14.0)	−35.7 (17.6)	1,380	.80
Wetness *SD*	21.7 (6.3)	23.2 (8.4)	1,226	.52
Brightness (variations in soil background reflectance)	43.1 (8.5)	46.0 (15.8)	1,086	.14
Brightness *SD*	19.6 (6.2)	21.8 (9.6)	1,157	.30
Disturbance index (km^2^)	0.2 (0.3)	0.1 (0.4)	1,523	.16
Class metrics (km^2^)				
Forest patch size	0.81 (0.69)	0.94 (0.91)	1,962.5	*<.0001*
Total forest area	8.29 (2.82)	7.533 (3.03)	2,211	*<.0001*
Mangrove patch size	3.73 (1.88)	4.03 (2.90)	1,374.5	.83
Total mangrove area	8.32 (2.34)	8.66 (2.96)	1,223.5	.51
Total coastal vegetation	0.73 (0.59)	0.48 (0.42)	1,691	*.03*
Number of forest patches	2.5 (3.2)	2.8 (3.5)	1,264	.65
Forest patch size CV	0.43 (0.47)	0.65 (0.49)	1,409	.68
Number of mangrove patches	7.9 (5.4)	7.6 (5.0)	1,351	.94
Mangrove patch size CV	1.69 (0.51)	1.51 (0.70)	1,584.5	.14
Number of coastal vegetation patches	25.2 (14.5)	19.1 (12.6)	1,660.5	.06
Coastal vegetation patch size CV	1.19 (0.95)	0.83 (0.51)	1,608	.11
Number of lagoons	4.4 (2.2)	3.0 (1.7)	1,579.5	*.00*
Mean shape index of forest	1.6 (0.3)	1.5 (0.3)	1,484.5	.38
Mean shape index of mangrove	1.7 (0.3)	1.6 (0.3)	1,363.5	.87
Mean shape index of coastal vegetation	1.2 (0.1)	1.1 (0.1)	1,670	.05
Landscape metrics				
Mean shape index of all land covers	1.3 (0.1)	1.3 (0.1)	1,637	.07
Shannon's diversity index	0.9 (0.2)	0.8 (0.3)	1,534.5	.25
Mean proximity index	29.4 (14.5)	27.3 (26.1)	1,566	.18
Landscape shape index	4.0 (1.0)	3.6 (0.9)	1,629.5	.07
Patch richness	3.9 (0.9)	3.9 (0.9)	1,369.5	.84

Note

Mean and standard deviation of nesting sites and random plots are presented. Statistically significant differences are in italics.

Five components accounted for 47% of the cumulative variance (Table 2). The 14 metrics with the highest absolute loading per component were used for the logistic model analysis and ecological niche modeling (Table 2). A high negative loading of wetness and high positive loadings of wetness standard deviation, brightness and its standard deviation characterized the first component. It described a gradient from areas with little moisture of soil and vegetation, large variability of moisture and shine among plots toward areas completely flooded. The second component indicated nests are located into a gradient from areas with regular‐shaped landscape and absence of coastal vegetation to those with a large amount of coastal vegetation patches and a very irregularly shaped landscape. The third component showed a great variability of mangrove patch size with regular shapes in homogeneous vegetation. The fourth component indicated a gradient from many forest patches, large areas of coastal vegetation with irregular patches, and great variation in size toward sites dominated by mangrove swamps with a few coastal vegetation proportions. Finally, the fifth component indicated the influence of high diversity of landscape and patch richness.

**Table 2:** Overall principal component analysis for the Cuban Black Hawk nesting sites and random plots during the 2012–2013 nesting seasons

Metric	Component loading				
	D1	D2	D3	D4	D5
Eigenvalue	4.8	4.1	3.5	2.7	1.9
% Total variance	10.3	10.0	7.3	10.6	8.6
Cumulative %	10.3	20.3	27.5	38.1	46.7
Shannon's diversity index	0.0	0.4	0.1	0.3	*0.7*
Landscape shape index	−0.1	*0.8*	0.1	0.1	0.3
Patch richness	0.0	0.0	0.0	0.0	*0.9*
Wetness	*−0.8*	0.0	−0.2	−0.1	0.4
Wetness (*SD*)	*0.7*	0.1	0.1	0.1	0.1
Brightness	*0.8*	0.0	0.0	−0.1	−0.1
Brightness (*SD*)	*0.9*	−0.1	0.1	0.0	0.1
Number of forest patches	0.1	−0.1	0.1	*0.7*	−0.3
Mangrove patch size coefficient of variance	0.1	0.4	*0.7*	0.1	0.0
Mean shape index of mangrove	−0.1	0.2	*−0.9*	−0.1	0.1
Total coastal vegetation area	0.0	0.3	0.1	*0.9*	0.1
Number of coastal vegetation patches	0.0	*0.9*	−0.2	−0.1	0.0
Coastal vegetation patch size coefficient of variance	0.1	0.0	0.1	*0.8*	0.1
Mean shape index of coastal vegetation	−0.1	−0.2	0.1	*0.7*	0.1

Note

D = principal components. Component loadings >0.7 are shown in italics.

Among all competing models built with combinations of the first five components (Table 3), the best logistic model (−2Log(likelihood) = 23.8; AIC = 37.3; Schwarz's Bayesian Criterion = 79.1) included five combinations of variables (Table 3). When including in modeling only the most weighted variables of the PCA axes (≥0.8) having a biological explanation, significant combinations were as follows: wetness and soil brightness in interaction with the landscape shape/number of coastal vegetation patches, and mangrove shape in interaction with Shannon's diversity index/patch richness (Table 4). Model‐averaged parameter estimates showed that the probability of finding a nest was strongly influenced by the presence of sites with low soil–vegetation moisture and low soil reflectance, in a landscape with a low shape complexity of patches (regular shape), and few patches of coastal vegetation. In addition, this probability was also influenced by areas with similar mangrove patch size and shape (circular patches) in homogeneous landscapes (Table 4). The logistic model had a significant probability on the log ratio (*χ* ^2^ = 47.6; *p* < .0001), and a high percentage of correct classification for nest presences (87%) and pseudo‐absences (90%).

**Table 3:** Ranking of candidate models distinguishing the Cuban Black Hawk nesting sites and random plots during the 2012–2013 nesting seasons

No. of variables	Variables	−2 Log(likelihood)	Pr > LR	AIC	ΔAIC	SBC Schwarz	*df*	Residual deviance
1	D3*D5	50.34	0.00	56.34	19.08	74.05	49	50.17
2	D1*D2/D3*D5	36.82	0.00	44.82	7.56	68.43	46	35.44
3	D1*D2/D2*D4/D3*D5	30.10	0.00	40.10	2.84	69.61	44	25.98
4	D1*D2/D1*D5/D2*D4/D3*D5	27.55	0.00	39.55	2.29	74.96	43	22.83
5	D1*D2/D2*D3/D2*D4/D3*D4/D3*D5	23.75	0.00	37.26	—	79.07	41	13.27

Note

Null deviance = 72.546. D, Variables in the regression were components (D) of a principal component analysis.

**Table 4:** Model‐averaged parameter estimates of competing conditional logistic regression models distinguishing the Cuban Black Hawk nesting sites and random plots during the 2012–2013 nesting seasons

Parameter (most weighted variables per components of the PCA)	Estimate	*SE*	Wald chi‐square	Pr > *χ* ^2^
Intercept	−3.6	1.6	5.3	0.02
Wetness and soil brightness and its standard deviation * landscape shape/number of coastal vegetation patches (D1*D2)	−8.7	3.8	5.4	0.02
Landscape shape/number of coastal vegetation patches * mangrove patch size coefficient of variance/mangrove shape (D2*D3)	−2.7	1.4	3.4	0.06
Landscape shape/number of coastal vegetation patches * total coastal vegetation area/coastal vegetation patch size coefficient of variance/coastal vegetation shape (D2*D4)	1.8	0.9	3.2	0.07
Mangrove patch size coefficient of variance/mangrove shape * total coastal vegetation area/coastal vegetation patch size coefficient of variance/coastal vegetation shape (D3*D4)	2.9	1.5	3.6	0.06
Mangrove patch size coefficient of variance/mangrove shape * Shannon's diversity index/patch richness (D3*D5)	−4.1	1.7	5.9	0.01

Note

Variables in the regression were components (D) of a principal component analysis (PCA).

### 3.4 Ecological niche model

**Figure 4:**
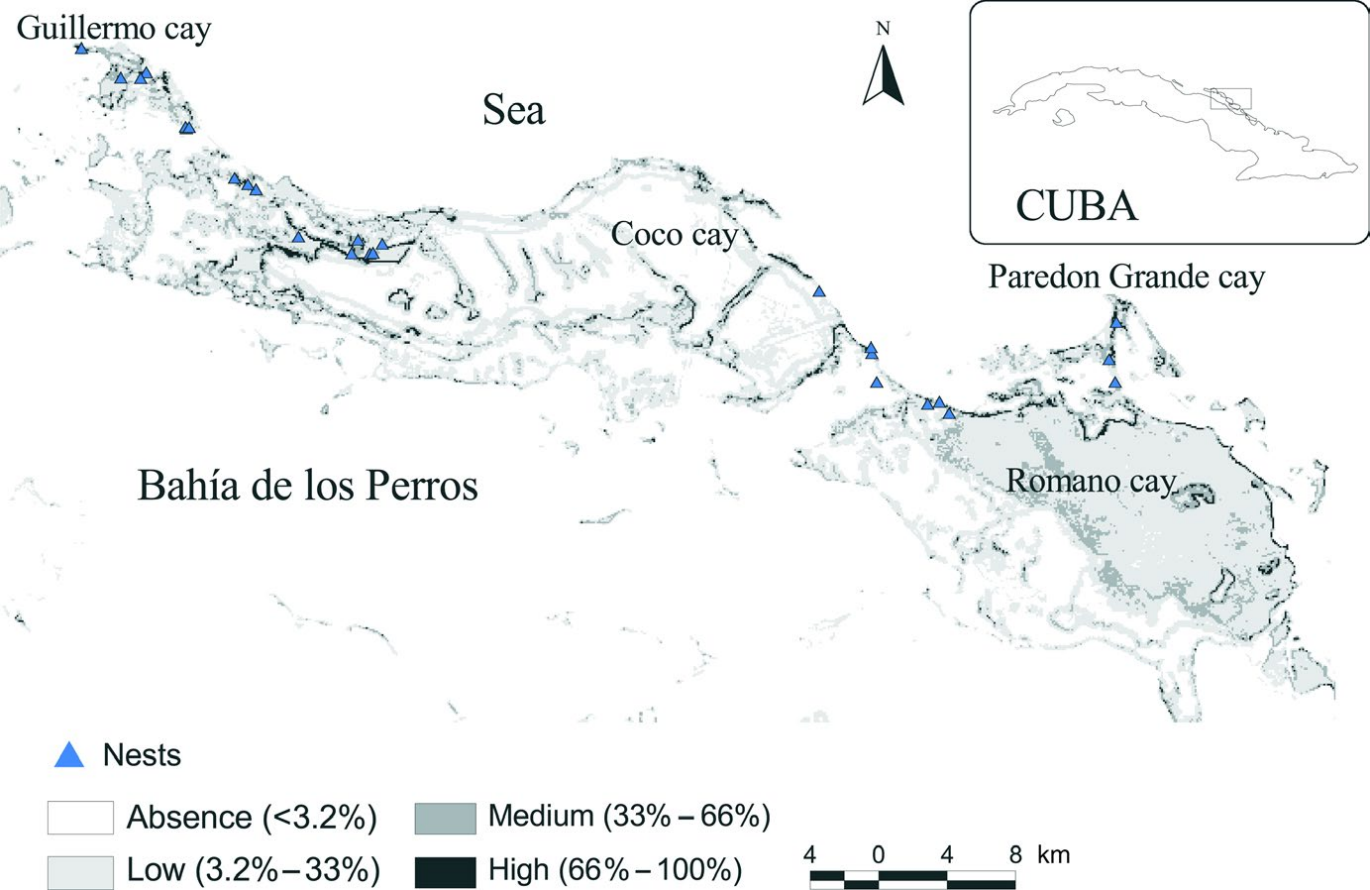
Geographic potential distribution of nests of the Cuban Black Hawk in Ciego de Ávila, Cuba. Percentages refer to the favorable condition probabilities

The potential distribution of Cuban Black Hawk nests indicated they are more probably located near the coastline, mainly concentrated in the western and eastern regions of the archipelago (Figure 4). The model predicted a potential distributional area of 863 km^2^ for the endemic hawk; 64% (556 km^2^) of this area is a narrow region located mainly in the mangrove swamp and corresponds to the area with suitable conditions for nesting of the Cuban Black Hawk. Remarkably, the area with a high favorable value is about 2% of the potential distribution, whereas the less favorable area exceeds 50% (Table 5). The remaining 36% of the potential distribution area was considered as unsuitable habitat (e.g., representing absences) after fitting the threshold value (0.032).

**Table 5:** Distributional nesting area of the Cuban Black Hawk in Ciego de Ávila, Cuba, based on ecological niche modeling

Distributional probability category	Area (km^2^)	Percentage of the total area	Nesting area inside PA (km^2^)	Percentage of PA per category
Absence (<3.2%)	307.5	35.6	117.7	38.3
Low (3.2%–33%)	437.2	50.6	98.5^a^	22.5
Medium (>33%–66%)	99.3	11.5	24.1^a^	24.3
High (>66%–100%)	19.3	2.2	6.1^a^	31.6
Total	863.3	100	246.4	—

Note

Percentages in the first column refer to the probabilities of having favorable condition.

a Nesting area extension (km^2^) under protection by the National System of Protected Areas (PA).

AUC scores indicated that Maxent had a high performance (training AUC = 0.9, test AUC = 0.8). The mean value for AUC ratio at 0.05 was significant 1.7 ± 0.12 (1.19–1.95), *p* < .0001, and for partial AUC was 0.84. Maxent predicted all sites in the cays where nests were found as having habitat with high suitability for the species (Figure 4). There were no omission errors for training or test data.

**Figure 5:**
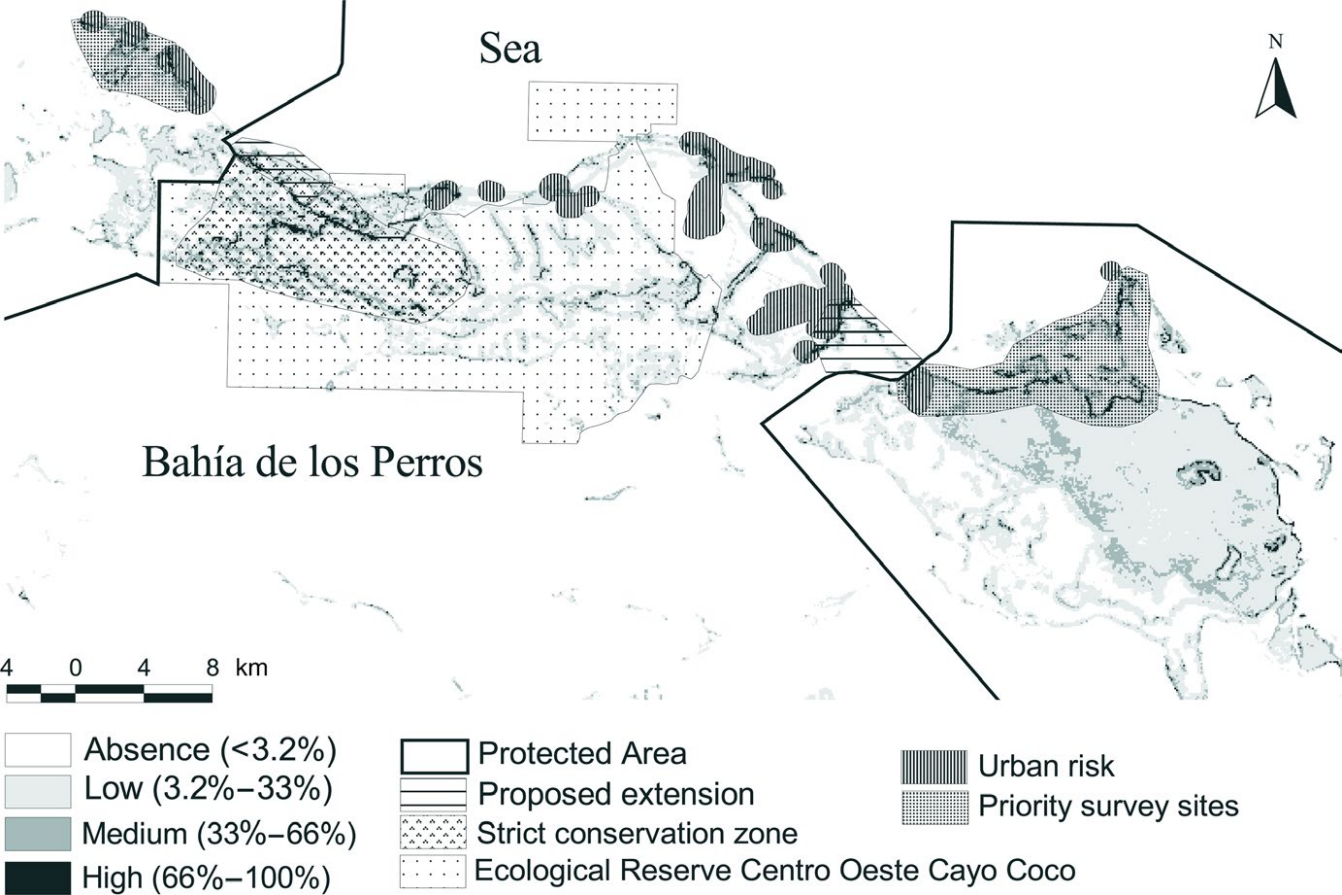
Recommendations for protected area extensions and priority survey sites based on the geographic potential distribution of nests of the Cuban Black Hawk at Ciego de Avila, Cuba. BV, Managed Resource Protected Area Buenavista; C‐OCC, Ecological Reserve Centro‐Oeste Cayo Coco; HCR, Managed Resource Protected Area Humedales de Cayo Romano

Nine (33%) nests were located within PAs while seven (27%) were close to or inside high‐risk zones. Ten percent and 6% of the area with high and medium probabilities of finding conditions similar to those in the sites where nests were registered, respectively, overlapped with the urban risk region. Similarly, 23% and 10% of the area with high and medium probabilities respectively, overlapped with the road risk region (Figure 5).

At present, there are three PAs officially protecting 23% of the Cuban Black Hawk predicted nesting distribution in the study region (Figure 5). Only 32% (6 km^2^) of the high suitable condition zones of the potential distribution have protection (Table 5). The highest percentage of protection (38%) corresponds to zones (117.7 km^2^) with <0.32 probability of favorable conditions (Table 5). To protect the largest possible extension of the geographic nesting potential distribution, we propose two feasible PA extensions: one to the northwest of the Ecological Reserve Centro‐Oeste Cayo Coco and the other to the east of the Managed Resource Protected Area Humedales de Cayo Romano (Figure 5). With these PA extensions, we could officially protect all known nesting sites. Two regions are also recommended as priority survey sites in eastern and western PAs, taking into account the high number of nests and the high probabilities of suitable habitat conditions (Humedales de Cayo Romano and Buenavista, respectively). Finally, we suggest evaluating the official conservation zone of the Ecological Reserve Centro‐Oeste Cayo Coco (e.g., strict zone) and we propose a new conservation area in such a way to include all the nesting areas with high favorable conditions (Figure 5). With this proposal, we would strengthen the nest monitoring system and guarantee better conservation strategies for the species in the critical period of nesting and nestling rearing during breeding.

## 4 DISCUSSION

Nests of the Cuban Black Hawk were found mainly in mangrove swamp, where the species has been described as common inside coastal zones and lagoons (Ferrer‐Sánchez & Rodríguez‐Estrella, 2015). Pairs were tolerant to humans approaching their nests and to vehicular traffic; thus, we found several nests and sightings of individuals near roads contrary to many other raptors that are affected by roads (Martínez‐Abraín, Oro, Jiménez, Stewart, & Pullin, 2010). Commonly, raptors that nest in trees tend to place their nests farther away from roads compared to raptors nesting in cliffs, suggesting a higher vulnerability due to potential predators and human presence (Morán‐López, Sánchez Guzmán, Borrego, & Sánchez, 2006).

The tolerance to roads and human disturbance by Common Black Hawk *B. anthracinus*, a closely related species, has also been reported (Sadoti, 2012). It seems that Black hawk' species may tolerate some level of human disturbance like ranches, residences, roads; however, sustained human presence near breeding pairs may lead to nest abandonment, nonuse, and delayed nesting (Schnell, 1994). On the contrary, the Common Black Hawk in New Mexico nests more than 1,000 m away from a high traffic paved road (Duffy, 2012).

It is possible that behavioral differences could be related to local population differences in habituation to humans owing to higher or lower levels of exposure to human presence, thus reflecting regional historical differences between areas, such as differential human densities or traffic intensities (Martínez‐Abraín et al., 2010). It is also likely that Cuban Black Hawk evolved to this kind of tameness behavior in the island because no potential predators exist (Cooper, Pyron, & Garland, 2014). Tameness of island species has conducted them to precipitous declines and extinctions when human activity increases, so caution should be taken by decision‐making and managers for the Cuban Black Hawk.

### 4.1 Breeding performance

The Cuban Black Hawk laid eggs more frequently in May. The average clutch size is similar for all populations in Cuba (Wiley & Garrido, 2005). No studies on breeding success and productivity of the species have been made before, so no comparisons on breeding performance are possible. However, comparing with a Common Black Hawk population in southwestern New Mexico, the percentage of pairs that successfully fledged at least one young was similar to our findings (ca. 70%; Sadoti, 2008). *Buteogallus anthracinus* in Arizona had a relatively higher nesting success (78%, *n* = 168 nests from 1976 to 1994; Schnell, 1994). These populations showed variability in productivity between years likely as a function of food availability. Long‐term monitoring of nesting success and productivity should be made for Cuban Black Hawk.

### 4.2 Nest‐site selection

Landscape elements of the nesting site can be separated into two groups in order to explain differences between nest‐site and random plots, group 1: distance to human activity, distance to roads, density of roads, distance to coastline, distance to patch edge, and group 2: forest patch size, total forest area, total coastal vegetation areas, and number of lagoons. A shorter distance to human activity, to roads, and a high density of roads at nest sites could be related to the low human density in the cays, and there could be similarities in the areas that developers look for hotels' constructions. In addition, the tourist area in the cays coincides with beaches, mangroves, and lagoons that are the preferred habitats of this hawk. The rest of the cays is covered by extensive forests and flooded areas where no nesting of the species has been observed. Therefore, there is a coincidence between nesting habitats and tourist development, which does not imply that hawks are preferring to breed near hotel infrastructures or paved roads. Shorter distances to the coastline and high number of lagoons in the site around nests are related to short distances to foraging areas and better access to a greater availability of prey. Due to these landscape features, it is likely that selection of nesting sites by Cuban Black Hawk is based on having short distances to foraging areas and a high availability of suitable foraging habitats near nests, as suggested for raptors in general (Newton, 1998). The availability of suitable nesting habitat is an important driver in the species nesting distribution and population productivity (Kendall, Rubenstein, Slater, & Monadjem, 2018; Wilson et al., 2018).

Several variables that differed between nesting sites and random plots were related to the total area of forest and coastal vegetation. A smaller forest patch size around nesting sites is related to isolated patches used as perch and vigilance sites for hunting where birds can fly and maneuvre (Wiley & Garrido, 2005). A greater total forest area indicates preferences to less modified areas in the landscape where habitat amount in the landscape is dominated by nonmodified vegetation. Also, a greater area of coastal vegetation indicates a separation between the nesting sites and the tourist activity since the latter leads to the loss and degradation of a large area of this coastal vegetation. Thus, landscape composition around bird nests is a key factor for foraging and also because it could strongly influence nest predation, a major cause of reproductive failure for many species (Chiavacci, Benson, & Ward, 2018).

Results of the logistic models identified a strong influence of a landscape with a low shape complexity of patches (regular shape), few patches of coastal vegetation, areas with similar mangrove patch size and shape (circular patches) in homogeneous landscapes. Circular homogeneous patches of mangroves in a landscape with low diversity and richness of native vegetation are closely related to the specialist habits of the Cuban Black Hawk.

Raptors seem to be dependent on environmental features related to landscape heterogeneity (e.g., Atuo & O'Connell, 2017; Campion, 2004; Martínez‐Ruiz & Renton, 2018). Conversely, the Cuban Black Hawk nesting sites were related to homogeneous landscapes highly explained by its specialized habits. Heterogeneous landscapes that are characterized by high patch densities and small patch sizes have been shown to benefit generalist species with detriment of habitat specialists (Devictor et al., 2008). Increases in configurational heterogeneity reduce large patches of homogeneous vegetation into smaller ones (Fahrig et al., 2011). If the reduction in minimum patch sizes for specialist species forces individuals to make use of multiple cover types, including suboptimal cover, they could be at a competitive disadvantage as compared to generalists (Bertrand, Burel, & Baudry, 2016). This will negatively affect habitat specialists that often rely on large patches of distinct cover types for population persistence. Maintaining large patches of distinct cover types will benefit specialist species (Atuo & O'Connell, 2017).

Landscape features surrounding nests should provide good habitat quality and contribute to improve reproductive success and fitness (Michel, Naef‐Daenzer, Keil, & Grüebler, 2017). In fact, the high reproductive success we found for the Cuban Black Hawk in mangrove habitat could reflect the high‐quality conditions of nesting areas. Nevertheless, studies that measured variables at landscape scales are more likely to detect effects of landscape variation and fragmentation on avian nesting success (Stephens, Koons, Rotella, & Willey, 2004). For instance, the extent of landscape explains best for variation in nesting success in grouse females and her brood, which suggests that landscape‐scale factors may override local factors such as track size and distance from edge (Kurki, Nikula, Helle, & Linden, 2000). The amount of high‐quality habitat patches, their distribution, and the accessibility of resources therein play a key role in regulating habitat effects on reproductive success of animals (Benton, Vickery, & Wilson, 2003). Not only the availability of different resources but also their position relative to each other is expected to affect the linked energy budget and the reproductive success of individuals (Michel et al., 2017).

Cuban Black Hawk nesting sites close to areas with human activity is similar with locations of nests of *B. anthracinus* found in Cliff‐Gila Valley, New Mexico (Sadoti, 2012), and with nesting areas of many diurnal raptors in Mediterranean landscapes as well (Campion, 2004). It is likely that human activities were not a strong predictor in our models of nesting site preferences because the low levels of human activity near the suitable habitat in Cuba compared to levels observed elsewhere (Sadoti, 2012). Also, there are two factors to take into account: (a) the limited area of the cays and (b) the suitable nesting habitat is restricted close to coastal zones that coincide with the tourism development. It is important to highlight that the spatial and temporal variation of many significant variables associated with nesting sites and their availability have been altered by human action, and can even increase in the near future. Increasing human disturbance and climate change will modify nesting site features and their availability, especially in Neotropical islands for rare, endemic, and specialist species like the Cuban Black Hawk (Ferrer‐Sánchez & Rodríguez‐Estrella, 2015). Also, it is important to understand the relative effects of landscape habitat loss, and habitat fragmentation, because they may change with the landscape limit size considered because the multiple (and potentially conflicting) ecological processes that are influenced by landscape structure occur at different spatial scales (e.g., dispersal, predation, foraging, reproduction) (Smith, Fahrig, & Francis, 2011).

### 4.3 Modeling approach and conservation strategies

Landscape scale perspective including several methods as principal component analysis, logistic regression (generalized linear models), and ecological niche modeling increased the information of Cuban Black Hawk nesting areas despite errors and uncertainty inherent to distribution models because data may not include all environmental, ecological, and historical factors that affect species distributions (Carvalho, Brito, Pressey, Crespo, & Possingham, 2010; Guisan & Zimmermann, 2000); thus, there might be some degree of uncertainty in the environmental variables used to generate models.

Our model can be used both for identifying potential nesting areas and nests' distribution and to design conservation strategies, showing new areas in which conservation would maximize the logistics and money investment for the nesting sites protection. Model showed that high suitable environmental conditions (2% of the distribution) in an unoccupied nesting habitat (e.g., low shape complexity of patches and few patches of coastal vegetation, similar mangrove patch size and shape, homogeneous landscapes) exists inside the potential distribution of nests. This means, that suitable habitat for the species is not yet saturated by breeding pairs. This is particularly important for a rare species because conservation planning and species recovery can consider many different potential areas to manage nesting sites, and to identify viable population's sites. Information on prey availability is also needed to strengthen the recommendations.

Only 32% of these suitable areas are protected by PAs. A common strategy for the conservation of nesting areas would be the design of new PAs networks aiming to preserve the biological uniqueness *in situ* (Possingham, Wilson, Andelman, & Vynne, 2006). However, when time, money, and resources are limited, it is more efficient to build upon existing PAs than to create new ones (Fuller, Munguia, Mayfield, Sanchez‐Cordero, & Sarkar, 2006), but the creation of new PAs should not be discarded. Therefore, current surveillance and monitoring systems can be strengthened for the species. Better proposals to the governmental agencies can be made on the need to extend the limits of the current PAs, and relocate the areas of strict conservation (e.g., preservation), in order to preserve most important sites (i.e., high nest concentration and suitable environmental conditions).

Habitat protection is of prime importance for maintaining raptor populations. The conservation of the Cuban Black Hawk will depend of strategies at the landscape level, including the design of circular homogeneous patches (regular shape) of mangroves, coastal vegetation, and coastal forests in a landscape with low diversity of vegetation. Protected areas can help by reducing or preventing habitat loss and degradation but it is urgently needed to protect nesting sites that are not currently included in the PAs network.

Plans of tourism facilities development in the short to medium term will certainly produce habitat changes that will decrease coastal vegetation, mangroves, and forest extension. If habitat changes continue increasing on the island of Cuba, affecting the remaining suitable habitat, we predict this species will become rarer and even face extinction in the medium term. If the scarce coastal vegetation is lost, and the small mangrove patches disappear, areas and habitats that are selected, and preferred by the species, and that increase the breeding success, will be eliminated.

Our methodological approach at the landscape scale could be useful to study rare and endangered species in other regions, particularly on islands where more vulnerable species occur. Ecological niche modeling can certainly strengthen the management and conservation actions not only for rare raptor species, but also for 238 endemics and threatened birds of the Neotropic that have small populations size, restricted distributions, and that are often habit specialists. Establishing predictive maps of nesting sites and determining the priority areas for conservation, because they have the highest values of preference (habitat selection), and therefore more suitable habitats, and where greater reproductive success would be expected, make it possible to propose a change in the design of PAs in a region, and even the expansion of the area for conservation management. Priority surveys inside and outside PAs can be established in order to have a greater impact on improving the species conservation status at broader scales.

## 5 CONCLUSIONS

This is the first study to present more deeply and detailed information on the breeding parameters of the Cuban Black Hawk. The landscape ecology approach allowed to identify the variables of the habitat that seems to be important for nesting of the species at a scale greater than local. Local characteristics of nests and habitat type showed that the nesting habitat of this endemic hawk is in mangrove swamp, having low trees close to the coastline, with low soil–vegetation moisture. It is important to note that the Cuban Black Hawk nesting habitat seems to be in good quality condition because the productivity and reproductive success of the species were high, similar to those of closely related species in undisturbed areas. Undisturbed mangrove swamp near the coastline was important for the Cuban Black Hawk nesting but only a very small proportion of the total area was highly suitable for the species. Furthermore, only a small fraction of the nests and suitable habitats are located in protected areas. These findings should have strong conservation implications for the species that policymakers need to include in the species management plans, being an endemic specialist with a narrow distribution in Cuba.

Models built to predict potential nesting sites and nest‐site selection allow to develop adequate conservation inferences (e.g., determine PA extensions and priority survey sites) and also to target the limited economic resources for conservation in an efficient way. Finally, we identified highly suitable areas for the species that are unprotected. We suggest expand the protected areas in order to include all known nesting sites under legal protection. Based on our findings, we propose that research on nesting habitat selection of top‐order predators, particularly of rare Neotropical raptors should be conducted at the landscape scale since landscape factors likely influences the breeding‐site selection.

## CONFLICT OF INTEREST

None declared.

## AUTHOR CONTRIBUTIONS

RRE and YFS conceived the ideas and designed the methodology; YFS, RRE, and MAM‐M analyzed the data; YFS and RRE led the writing of the manuscript; MAM‐M also contributed ideas during the process. All authors contributed critically to the drafts, ensured the accuracy and integrity of any part of the work, and gave final approval for publication.

## Data Availability

Our spatial data and environmental characteristics used in modeling and supporting our results are in the public repository Dryad, Dataset, https://doi.org/10.5061/dryad.kwh70rxzv.
