# Supplementary material for: Improving conservation strategies of raptors through landscape ecology analysis: The case of the endemic Cuban Black Hawk
Source: Ecol Evol. 2019 Nov 27;9(24):13808–23. doi: 10.1002/ece3.5815 (PMC6953671; doi:10.1002/ece3.5815)
Supplement: Supplementary file 1 [file ECE3-9-13808-s001.docx]

Appendix S1. Fifty-eight landscape metrics tested in this study.

| **Metric name** | **Description** |
| --- | --- |
| Distance to road (m) | Distance from each nest to road |
| Distance to human activity (m) | Distance from each nest to human activity |
| Distance to coast (m) | Distance from each nest to coastline |
| Distance to patch edge (m) | Distance from each nest to patch edge |
| Density of roads | Total length of roads per unit area |
| NDVI | Normalized difference vegetation index |
| Standard deviation of NDVI | Standard deviation of the normalized difference vegetation index indicating variability |
| OSAVI | Optimized soil-adjusted vegetation index |
| Standard deviation of OSAVI | Standard deviation of the optimized soil-adjusted vegetation index indicating variability |
| Wetness | Interrelationship of soil and canopy moisture |
| Standard deviation of wetness | Standard deviation of wetness (interrelationship of soil and canopy moisture) |
| Brightness | Variations in soil background reflectance |
| Standard deviation of brightness | Standard deviation of the soil background  reflectance |
| Disturbance index | Area (km^2^) of urban zone |
| Forest patch area | Size of the patches of forest |
| Total forest area | Total amount of forest area |
| Mangrove patch area | Size of the patches of mangrove |
| Total mangrove area | Total amount of mangrove area |
| Coastal vegetation patch area | Size of the patches of coastal vegetation |
| Total coastal vegetation area | Total amount of coastal vegetation area |
| Number of forest patches | Equals the number of forest patches |
| Forest patch size coefficient of variance | Indicate the variability in the size of all forest patches |
| Number of mangrove patches | Equals the number of mangrove patches |
| Mangrove patch size coefficient of variance | Indicate the variability in the size of all mangrove patches |
| Number of coastal vegetation patches | Equals the number of coastal vegetation patches |
| Coastal vegetation patch size coefficient of variance | Indicate the variability in the size of all coastal vegetation patches |
| Number of lagoons | Equals the number of lagoons in the area |
| Patch density | Number of patches per area |
| Largest patch index | Percentage of total area occupied by the largest patch |
| **Shape Metrics** | |
| Mean shape index of all habitats | Equals 1 when all patches are circular; increases with complexity of patch shapes; independent of patch size |
| Mean shape index of forest | Equals 1 when all forest patches are circular; increases with complexity of patch shapes; independent of patch size |
| Mean shape index of mangrove | Equals 1 when mangrove patches are circular; increases with complexity of patch shapes; independent of patch size |
| Mean shape index of coastal vegetation | Equals 1 when coastal vegetation patches are circular; increases with complexity of patch shapes; independent of patch size |
| Perimeter–area ratio | Patch shape complexity measure that measures perimeter per area |
| Forest fractal dimension index | Measure of complexity of forest patch shape that approaches 1 for simple shapes and 2 for complex shapes |
| Mangrove fractal dimension index | Measure of complexity of mangrove patch shape that approaches 1 for simple shapes and 2 for complex shapes |
| Coastal vegetation fractal dimension index | Measure of complexity of coastal vegetation patch shape that approaches 1 for simple shapes and 2 for complex shapes |
| Perimeter–area fractal dimension of forest | Forest patch shape complexity measure, which approaches 1 for shapes with simple perimeters and 2 for complex shapes |
| Perimeter–area fractal dimension of mangrove | Mangrove patch shape complexity measure, which approaches 1 for shapes with simple perimeters and 2 for complex shapes |
| Perimeter–area fractal dimension of coastal vegetation | Coastal vegetation patch shape complexity measure, which approaches 1 for shapes with simple perimeters and 2 for complex shapes |
| Area-weighted mean patch fractal dimension | At the class and landscape levels by weighting patches according to their size, similar to the area-weighted mean shape index. AWMPFD approaches 1 for shapes with very simple perimeters, such as circles or squares, and approaches 2 for shapes with highly convoluted, plane-filling perimeters |
| **Diversity and Interspersion Metrics** | |
| Shannon’s diversity index | Equals minus the sum of the proportional abundance of each patch type multiplied by the ln of that proportion |
| Simpson’s diversity index | Diversity measure, which equals 1 minus the sum of the squared proportional abundance of each patch type |
| Modified Simpson’s diversity index | Diversity measure, which equals minus the ln of the sum of the squared proportional abundance of each patch type |
| Shannon’s evenness index | Diversity measure, which considers only evenness of patch sizes, not the number of patches |
| Simpson’s evenness index | Diversity measure, which considers only evenness of patch sizes, not the number of patches |
| Modified Simpson’s evenness index | Diversity measure, which considers only evenness of patch sizes, not the number of patches |
| Patch richness | Equals the number of patch types |
| Relative patch richness | Percentage of present patch types out of all categories |
| Patch richness density (no./100 ha) | Equals the number of patch types (i.e. land cover categories) per 100 ha |
| Mean proximity index | Considers size and proximity of all patches with the same land cover type inside a specified search radius |
| Similarity index | Considers size and proximity of patches within a search radius, weighted by their similarity to the focal patch |
| Euclidean nearest neighbor distance | minimum edge to edge distance to the nearest neighboring patch of the same type |
| Contagion index | Measure of the aggregation of the land cover classes |
| Interspersion and juxtaposition ind. (%) | Measure of evenness of patch adjacencies, equals 100 for even and approaches 0 for uneven adjacencies |
| **Landscape metrics** | |
| Landscape shape index (LSI) | Ratio of the total edge to the minimum total edge |
| Edge density | Total length of edge per unit area |
| Contiguity index | Equals 0 for a one-pixel patch and approaches 1 as patch contiguity, or connectedness increases |
